# Short Comings of the Journal

**Published:** 1880-08

**Authors:** 


### SHORT-COMINGS OF THE JOURNAL.

The last two or three issues of the JOURNAL have been tardy, so much so, indeed, that subscribers, in some instances at least, have concluded that their names had been overlooked by the mailing clerk. This has been the result of overcrowded business in the publishing house not contemplated at this season of the year. We hope in future this will not occur, at any rate to the extent of our recent failures.

Failures to receive the JOURNAL occasionally occur, from causes not attributable to the publisher, and sometimes through oversight a name fails to be indorsed.

Subscribers will please notify the editor or publisher of such failure promptly, so that we may be able to fill the deficiency before the edition is exhausted. Nothing, we know, is more annoying than such delinquency, and we will seek, in future, not only to use all diligence to prevent failures, but afford correction at the earliest possible moment.

ADVICES from Europe and Smyrna make it appear probable that befor the end of the year the price of opium will be very high. A combination of wealthy speculators has been formed to purchase and hold the crop, and it is not unlikely that East India opium may come into our market.—*American Practitioner.*

